# RSPO3 antagonism inhibits growth and tumorigenicity in colorectal tumors harboring common Wnt pathway mutations

**DOI:** 10.1038/s41598-017-15704-y

**Published:** 2017-11-10

**Authors:** Marcus M. Fischer, V. Pete Yeung, Fiore Cattaruzza, Rajaa Hussein, Wan-Ching Yen, Christopher Murriel, James W. Evans, Gilbert O’Young, Alayne L. Brunner, Min Wang, Jennifer Cain, Belinda Cancilla, Ann Kapoun, Timothy Hoey

**Affiliations:** OncoMed Pharmaceuticals, Inc. 800 Chesapeake Drive, Redwood City, CA 94063 USA

## Abstract

Activating mutations in the Wnt pathway are a characteristic feature of colorectal cancer (CRC). The R-spondin (RSPO) family is a group of secreted proteins that enhance Wnt signaling and RSPO2 and RSPO3 gene fusions have been reported in CRC. We have previously shown that Wnt pathway blockers exhibit potent combinatorial activity with taxanes to inhibit tumor growth. Here we show that RSPO3 antagonism synergizes with paclitaxel based chemotherapies in patient-derived xenograft models (PDX) with RSPO3 fusions and in tumors with common CRC mutations such as APC, β-catenin, or RNF43. In these latter types of tumors that represent over 90% of CRC, RSPO3 is produced by stromal cells in the tumor microenvironment and the activating mutations appear to sensitize the tumors to Wnt-Rspo synergy. The combination of RSPO3 inhibition and taxane treatment provides an approach to effectively target oncogenic WNT signaling in a significant number of patients with colorectal and other intestinal cancers.

## Introduction

Wnt pathway mutations that activate signaling are present in virtually all colorectal tumors, emphasizing the Wnt pathway as the fundamental oncogenic driver in this disease. The commonly mutated targets include APC (adenomatous polyposis coli), β-catenin and RNF43^1,2^. Therapies targeting the Wnt pathway are in clinical development and have demonstrated activity against tumorigenic cancer cells^3,4^. The intestinal stem cell is responsible for tissue homeostasis and is characterized by expression of the cell surface marker Lgr5 (leucine-rich-repeat-containing G-protein-coupled receptor 5)^5^. Like the normal epithelium, intestinal cancers also contain Lgr5 expressing stem cells and these cells share properties of the normal Lgr5 stem cells^6–9^. The Wnt agonists R-spondins (RSPO), ligands for the LGR family including LGR4, LGR5, and LGR6, potentiate the Wnt pathway by interfering with the clearance of Wnt receptors from the plasma membrane^10,11^ and are essential in maintaining the LGR5^+^ stem cell^12^. RSPO2 and RSPO3 genomic rearrangements are an alternative genetic mechanism to up-regulate Wnt signaling^2,13^ and tumors harboring these translocations are highly sensitive to RSPO blocking antibodies^3,14^. Previous studies in breast, ovarian, lung and pancreatic tumors have shown that Wnt antagonists display unique combination activity with taxane chemotherapy^15^.

CRC is derived from adult intestine and shares similar features with that of the normal epithelium stem cell hierarchy. In the intestinal crypt, Wnt signaling maintains intestinal tissue homeostasis by stimulating adult multipotent intestinal stem cells (ISC). Lgr5 expression identifies the dominant ISC of the murine intestinal epithelium^5^. In CRC, LGR5 expressing tumor cells are a subset of tumor cells and share properties inherent to the ISC such as self-renewal capacity. Analysis of the local microenvironment, or stem cell niche, has identified mesenchymal stromal cells as an abundant source of Rspo3 and Wnt^16,17^ and targeted ablation of a subset of murine mesenchymal cells expressing *foxl1* in turn disrupts homeostasis of the intestinal crypt^17^. These mesenchymal cells are directly adjacent to the crypt stem cell and provide paracrine Wnt agonists including various Wnt ligands and Rspo3. Like the normal intestine, the CRC microenvironment contains supportive cell types such as tumor associated fibroblasts, endothelial cells, and suppressive immune cells. These cells are often recruited to the tumor site in response to tumor derived signals, and provide essential niche components, nutrients, and growth factors such as RSPO. In the intestine, Rspo signaling was found to be essential for promoting Lgr5 ISC self-renewal, while Wnt proteins primed the Rspo-Lgr5 axis by inducing Lgr5 expression^12^. Therefore, Rspo is an essential component of the stem cell niche in intestinal biology.

Through the common activating mutations discussed above, Wnt pathway signaling is widely dysregulated in CRC, as nuclear translocation of β-catenin is readily detected by immunohistochemistry and Wnt pathway target genes are upregulated^18^. Interestingly, even in the context of activating mutations, levels of Wnt pathway activity are not uniform in tumor cells and can be higher in certain regions of the tumor^18^.

**Figure 1:**
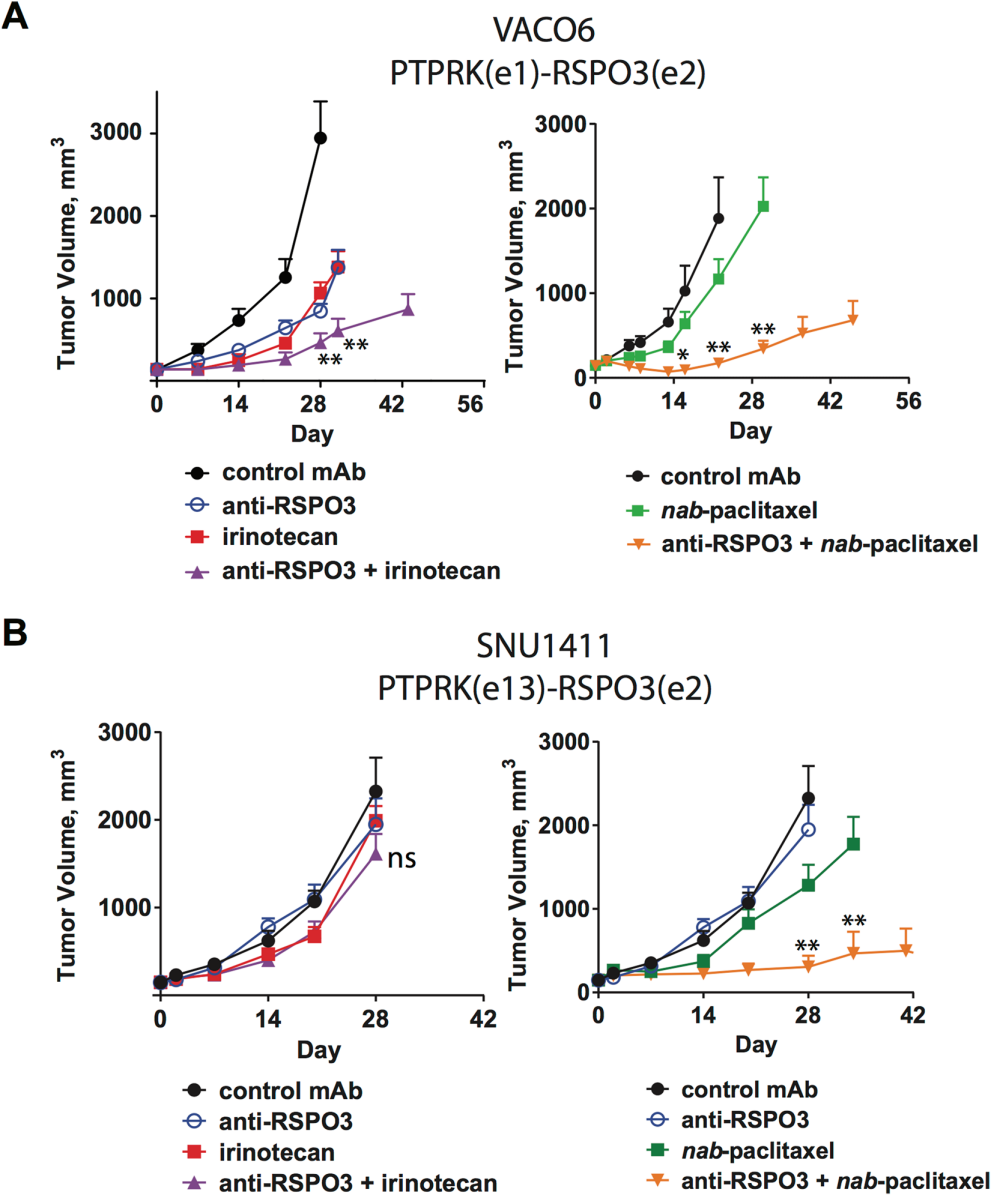
CRC with PTPRK-RSPO3 fusions respond to RSPO3-taxane treatment. (**A**) VACO6 cell line is responsive to irinotecan and *nab*-paclitaxel when combined with RSPO3 neutralizing antibody anti-Rspo3. (**B**) SNU1411 is non-responsive to anti-Rspo3, irinotecan, or anti-Rspo3 + irinotecan. SNU1411 can be sensitized to *nab*-paclitaxel when combined with anti-Rspo3. Anti-Rspo3 was dosed at 25 mpk Q2W on day 1, irinotecan was dosed at 10 mpk Q1W on days 3 and 10, and *nab*-paclitaxel was dosed at 25 mpk Q2W on day 3, 7–10 mice per treatment group. *p < 0.01 vs. *nab*-paclitaxel, **p < 0.001 vs. *nab*-paclitaxel, ns = not significant.

As an alternative to APC, β-catenin, or RNF43 mutations, malignant intestinal epithelium may activate the Wnt pathway by gaining PTPRK-RSPO3 translocations which results in expression of tumor-cell derived RSPO3^3,13,19^. Inhibition of RSPO3 with OMP-131R10 (anti-Rspo3; rosmantuzumab), a clinical-stage therapeutic antibody which binds to human and murine RSPO3, has demonstrated efficacy in CRC tumors harboring RSPO3 fusions^3^. This antibody also has activity in other epithelial tumor types overexpressing RSPO3, including lung and ovarian tumors which have RSPO3 overexpression driven by an unknown mechanism^3^. Anti-tumor activity in RSPO3 fusion containing CRC tumors has been reported with another RSPO3 neutralizing antibody^14^. Two CRC cell lines, VACO6 and SNU1411, have been identified which have PTPRK-RSPO3 fusions, overexpress RSPO3 and are sensitive to porcupine inhibition^20^. The VACO6 xenograft model demonstrates tumor growth inhibition when exposed to anti-RSPO3 alone or in combination with the topoisomerase inhibitor irinotecan or alternatively in combination with nab-paclitaxel (Fig. 1A). In contrast, the SNU1411 xenograft model was found to be resistant to anti-RSPO3 as a single agent or when combined with irinotecan (Fig. 1B). Both agents resulted in on-target gene modulation as anti-Rspo3 reduced *AXIN2*, *ZNRF3*, and *LGR5* expression in both VACO6 and SNU1411 (extended Fig. 1). We previously reported that antagonizing Wnt signaling with an antibody targeting multiple Frizzled proteins (vantictumab; OMP-18R5) or with a Frizzled8-Fc decoy receptor (ipafricept, OMP-54F28) synergized with the microtubule stabilizer paclitaxel in epithelial cancers lacking WNT pathway mutations^15^. Strikingly, we discovered that SNU1411 was highly sensitive to the combination of anti-RSPO3 and *nab*-paclitaxel even though it was resistant to anti-RSPO3 as a single agent or in combination with irinotecan (Fig. 1B). Thus, CRC is another example where Wnt pathway antagonism, either by biologics targeting the Frizzled receptors or their secreted agonists WNT or RSPO, displays synergistic anti-tumor activity in combination with a taxane and results in better combination activity than other types of chemotherapy.

**Figure 2:**
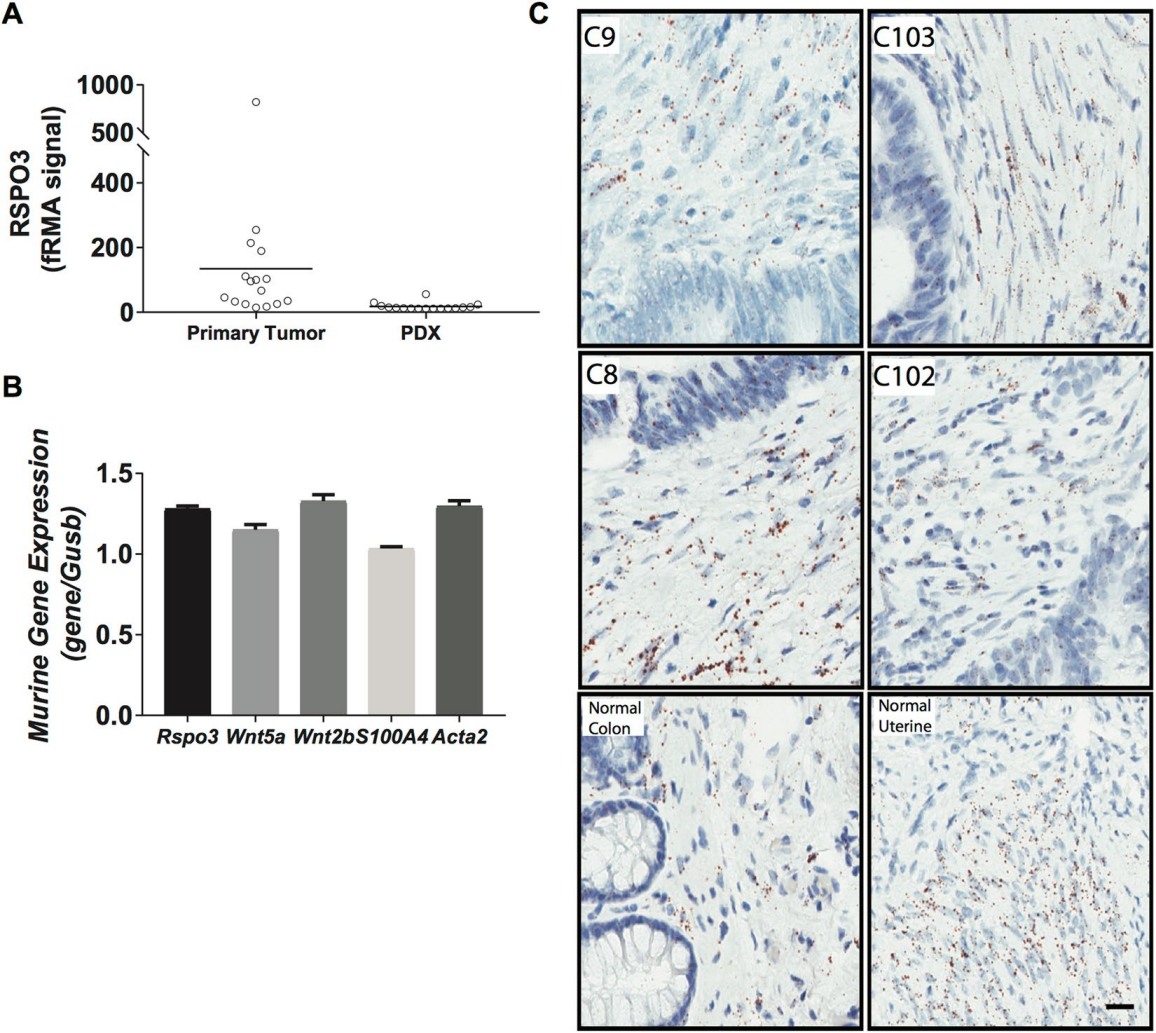
Colorectal cancers contain high levels of RSPO3 that is associated with the tumor microenvironment. (**A**) Microarray analysis of RSPO3 expression in primary CRC cancer and PDX tumors. Primary tumors (Tumor) express human RSPO3 while the expression of human RSPO3 is lost in the PDX. (**B**) qPCR of PDX models detects murine *Rspo3*, *Wnt*, and Tumor Associated Fibroblast markers, *S100a4* and *Acta2*. OMP-C40 tumor model whole tumor lysate, qPCR performed on Fluidigm Biomark. (**C**) Detection of RSPO3 by ISH using RNAscope. Primary patient colon tumor tissues used to create PDX tumors OMP-C9, OMP-C103, OMP-C88, OMP-C102, and normal human colon and uterine tissues were tested with the RNAscope® 2.5 VS Reagent kit, using probes for RSPO3. RSPO3-ISH is shown as brown dots and nuclei as blue. Scale bar = 20 μm.

Advances in translational research have led to the development of patient derived cancer models, and a model of particular translational relevance is the *in vivo* PDX (patient derived xenograft model)^21–23^. The PDX maintains the diversity of human epithelial cells, although non-epithelial cells such as those derived from mesenchymal or hematopoietic stem cells are not preserved^24^. In the PDX, mouse cells replace these human stromal cells and provide the microenvironment necessary to support tumor cell growth. Microarray analysis of primary CRC tumors has demonstrated readily detectable expression levels of human *RSPO3* while in the passaged CRC PDX models this expression of human *RSPO3* is no longer evident (Fig. 2A). In CRC PDX models, the expression of murine *Rspo3* in stromal cells of the tumor microenvironment was detectable by qPCR (Fig. 2B). Consistent with the qPCR data, *in situ* hybridization (ISH) analyses of primary human colon tumors indicated that *RSPO3* expression was primarily in stromal cells (Fig. 2C). Thus, CRC recapitulates the paracrine signaling mechanism found in the normal intestinal crypt^16,17^ where *RSPO3* is expressed in stromal cells adjacent to epithelial cells.

**Figure 3:**
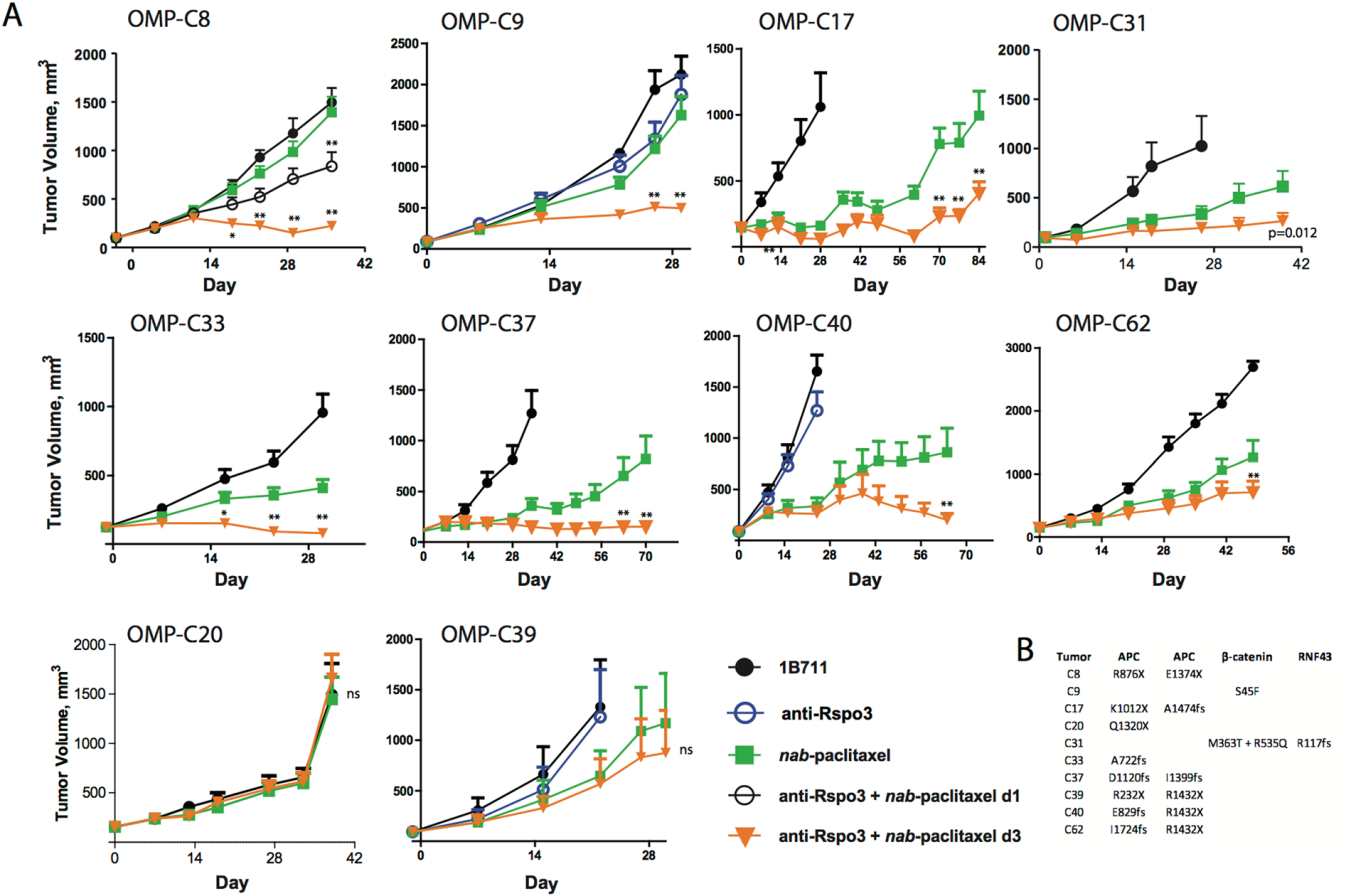
CRC PDX responds to Rspo3 antagonism in combination with taxane chemotherapy. (**A**) Eight of Ten CRC PDX models respond to taxane chemotherapy when anti-Rspo3 is dosed two days before *nab*-paclitaxel. anti-Rspo3 (25 mpk Q2W) dosed on day 1 and nab-paclitaxel (30 mpk Q1W) dosed on day 3 and day 10, in two week cycles is an active dosing regimen, n = 7–10 per treatment group, expressed as mean + SEM. *p < 0.01 vs. *nab*-paclitaxel, **p < 0.001 vs. *nab*-paclitaxel, ns = not significant. (**B**) CRC PDX models contain activating mutations in APC, β-catenin, or RNF43. Mutations were detected in tumor cells using Sanger sequencing.

Next, we sought to determine if Rspo3 antagonism in combination with a taxane could be effective in CRC tumors with common mutations in the Wnt pathway, such as APC or β-catenin. We examined the efficacy of anti-Rspo3 in combination with chemotherapy in ten CRC PDX models. Eight of these models contained inactivating mutations in APC while the remaining two contained mutations in either β-catenin or RNF43 (Fig. 3). None of these tumors harbor Rspo3 translocations. Generally, we did not observe single agent activity with anti-Rspo3 in these CRC tumors with low Rspo3 expression (Fig. 3 and data not shown). Importantly we consistently found that inhibition of tumor growth was observed with anti-Rpso3 in combination with taxanes. In contrast to the combination activity with taxanes in these types of tumors, combinations of anti-Rspo3 with 5-fluorouridine and/or with irinotecan were not effective (extended Fig. 2). Non-CRC tumors that express low/undetectable levels of human Rspo3 and are wild type for APC, β-catenin and RNF43 were not sensitive to the combination of anti-Rpso3 plus taxane chemotherapy (extended data Fig. 3). These data suggest mutations in the Wnt pathway sensitize CRC tumors to anti-Rspo3/taxane synergy. Pre-dosing anti-Rspo3 two days before nab-paclitaxel was more effective than simultaneous exposure, consistent with our findings with other Wnt pathway blockers^15^. Stronger responses were observed in five PDX models as interpreted as either stable disease or tumor regression while three models achieved statistically significant inhibition of tumor growth relative to chemotherapy alone (Fig. 3). Two models were entirely resistant to treatment, OMP-C20 and OMP-C39. These two tumors are not obviously different in their differentiation status when compared with sensitive tumors; future studies will interrogate the mechanisms underlying resistance to anti-Rspo3 treatment. The APC mutated tumors that were responsive to treatment all contained heterozygous mutations resulting in partial deletions in APC, suggesting an incomplete loss of function and retention of some ability to be modulated by signaling^25^. APC is a critical component of the β-catenin destruction complex and typical CRC mutations in APC reduce, but do not eliminate, its ability to sequester β-catenin and target it for proteolytic degradation^25^. These tumors appear to be addicted to the maintenance of high levels of Wnt activation, and Rspo3-LGR5 interactions directly potentiate this oncogenic pathway. Rather than being ligand-independent, our data suggest that CRC tumors harboring common Wnt pathway mutations can be viewed as hypersensitive to ligand dependent Rspo3 signaling.

**Figure 4:**
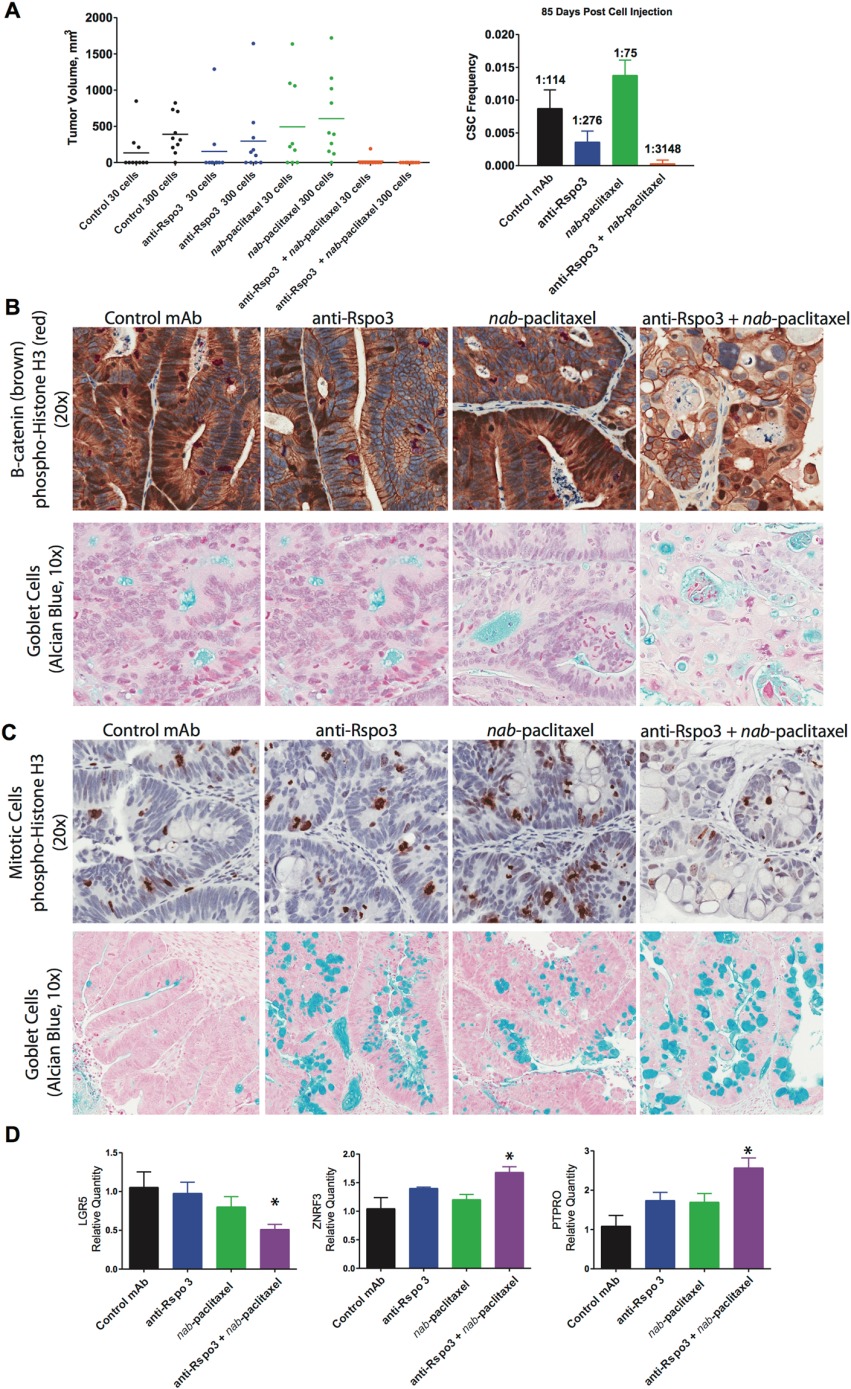
RSPO3-taxane regimen reduces CSC self-renewal properties by reducing β-catenin transduction and potentiating paclitaxel mediated mitotic blockade. (**A**) Limiting dose assay in OMP-C8 tumor model, from Fig. 3. The combination of anti-Rspo3 (25 mpk) on day 1 and *nab*-paclitaxel (30 mpk) on day 3 and day 10 in three-two week cycles reduces the cancer stem cell frequency as compared to *nab*-paclitaxel, by 40-fold. Tumors were serially passaged on day 38, seven days after the last treatment cycle, at a cell dose of 30 or 300 cells into recipient mice, 10 mice per cell dose and 20 mice per treatment group. On day 85 of the LDA, tumor take was determined and the CSC frequency was calculated. (**B**) Anti-Rspo3 + *nab*-paclitaxel reduced nuclear β-catenin expression, disrupted mitosis, and promoted the expansion of differentiated mucin producing goblet cells. OMP-C8 tumors were assayed for β-catenin expression (brown) and mitotic activity (red, PHH3) with nuclear counterstain (blue), 20x magnification. Goblet cells were detected by Alcian Blue counterstain (blue) with nuclei in purple, 10x magnification. (**C**) *Nab*-paclitaxel expands for mitotic cells while combination with anti-Rspo3 promotes for endoreduplication and the selection of differentiated goblet cells. From OMP-C9 study in Fig. 3. OMP-C9 tumors were assayed for mitotic activity (PHH3, brown) with nuclear counterstain (blue), 20x magnification. Goblet cells were detected by Alcian Blue counterstain (blue) with nuclei in purple, 10x magnification. D. RSPO3-taxane treatment reduces LGR5 expression and promotes suppression of Wnt signaling. Taqman qPCR, n = 2–4 per treatment group, expressed as mean + SEM, *p < 0.05.

As antagonism of Rspo3-LGR5 signaling is expected to impact tumorigenicity, we examined the ability of treated tumor cells to re-grow tumors after serial transplantation. In the OMP-C8 PDX model, an APC mutated tumor, three cycles of anti-Rspo3 + *nab*-paclitaxel treatment dramatically reduced the cancer stem cell frequency by 40-fold, as assayed by the limiting dilution assay (LDA) (Fig. 4A). The LDA is a serial transplantation assay performed in treatment naïve mice which directly measures the functional ability of tumor cells to form new tumors, without relying on expression of a particular stem cell biomarker. Other methods that are commonly utilized to assay stem cell properties include lineage tracing in transgenic mice. Targeted ablation of Lgr5 ISC identified a potential residual stem cell population as able to repopulate the Lgr5 stem cell compartment^26^, while in distant liver metastasis this repopulation was prevented^27^. Techniques have been developed to lineage trace human CRC organoid cultures, with similar findings^28^. Here, we demonstrate that the overall cancer stem cell population was dramatically reduced in tumor cells previously treated with anti-Rspo3 plus nab-paclitaxel.

In the majority of responsive tumors, an effect on reducing tumor growth was not evident until after the completion of the second treatment cycle. This suggests that the promotion of differentiated, chemo-sensitive tumor cells is a gradual process that requires successive exposure to the combination of anti-Rspo3 with paclitaxel. A potential explanation for this delayed activity is that a reserve cancer stem cell population exists within these tumors, as discussed above. These reserve tumorigenic cells may be non-cycling during the first treatment cycle, but become proliferative and Lgr5+, and thereby sensitive to continued anti-Rspo3 plus taxane treatment.

ISC and CSC self-renewal is associated with β-catenin signal transduction. We found our therapy reduced nuclear β-catenin expression (Fig. 4B) and promoted selection of differentiated cells (Fig. 4C). Anti-Rspo3 expanded the Alcian Blue reactive population, which in intestinal tissue is reliably used as a marker of differentiated secretory goblet cells (Fig. 4C). This occurred to a modest extent with anti-Rspo3 in combination with taxane treatment in OMP-C8 and much more dramatically in both single agent and combination groups in OMP-C9 (Fig. 4B and C). Combining anti-Rspo3 with paclitaxel resulted in an enlarged cellular morphology that closely resembles that of endoreduplication and mitotic catastrophe, while reducing tumor cell density. These histological phenotypes are similar to what we found with other Wnt pathway antagonists in combination with taxane chemotherapy, and it appears that the basis of synergy may involve both transcriptional and non-transcriptional mechanisms of Wnt signaling^15^. In the β-catenin mutated tumor OMP-C9, the combination treatment regimen reduced proliferation, tumor density, and resulted in numerous differentiated cells (Fig. 4C). Similar pharmacodynamic effects were seen in OMP-C8 and OMP-C9 including an increase in the expression of regulators of Wnt signaling, *PTPRO* and *ZNRF3*. In OMP-C9, *LGR5* expression was reduced (Fig. 4D) as has been observed in Rspo3 fusion CRC tumors after anti-Rspo3 treatment (Extended Fig. 1 and ref.^3^) These data demonstrate an effect of anti-Rspo3 treatment on Wnt target genes in the setting of APC or β-catenin mutated tumors.

These CRC models used here represent the spectrum of colorectal tumors as they harbor mutations in APC, β-catenin, RNF43 or the rare PTPRK-RSPO3 gene fusions. Tumor homeostasis was dysregulated by anti-Rspo3 plus taxane treatment and the combination enriched for differentiated colon tumor cells and reduced the frequency of cells with tumorigenic properties. Here we demonstrate that Rspo3 derived from the tumor microenvironment can be inhibited to induce a profound anti-tumor response. We have previously determined that paclitaxel treatment of tumors resulted in higher levels of mitotic cells with β-catenin expression, and that high levels of Wnt pathway components were associated with resistance to paclitaxel^15^. By blocking the Wnt-Rspo pathway with anti-Rspo3 together with mitotic blockade by paclitaxel, we have identified a strategy to sensitize CRC to taxane based chemotherapy. Based on our previous studies with Wnt pathway antagonists, the mechanisms of synergy of anti-Rspo3 and taxanes may involve cell cycle-related functions of β-catenin^15^, and promoting differention of CRC tumor cells to a secretory/goblet cell fate may also play a role. Our experiments provide the rationale for a broad utility of Rspo3 antagonism for the treatment of CRC that would include not only tumors with PTPRK-RSPO3 fusions, but also tumors with common Wnt pathway-activating mutations.

## Methods

### Ethics Statement

The animals used in this study were housed in a U.S. Department of Agriculture-registered facility in accordance with NIH guidelines for the care and use of laboratory animals. The study followed the guidelines set by OncoMed Pharmaceuticals’ Institutional Animal Care and Use Committee (IACUC). The experimental animal use protocols OMP3 and OMP6 relevant to these studies were approved by OncoMed IACUC committee. Additional accreditation to this facility was provided by the Association for Assessment and Accreditation of Laboratory Animal Care (AAALAC).

### Study Design

PDX tumor models were utilized in these studies. These models were established in immunocompromised mice and were never propagated *in vitro*. Each study contained a negative control antibody group consisting of a murine monoclonal antibody directed against dinitrophenol (1B711, ATCC). Anti-RSPO3 (OMP-131R10) is a human IgG1 monoclonal antibody that cross-reacts with human and murine RSPO3 and does not bind to RSPO1, RSPO2, or RSPO4^3^.

### *In vivo* Experiments with PDX models

NOD.CB17-Prkdc^scid^ (NOD/SCID) mice were purchased from Envigo or Charles River and maintained under specific pathogen-free conditions and provided with sterile food and water ad libitum. The mice were allowed to acclimate for several days prior to the PDX studies. Preparation of PDX tumor samples for *in vivo* studies were as described^15^. Treatments were initiated after randomization with inclusion of tumors at approximately 75 to 200 mm^3^. Subcutaneous tumor growth was measured with an electronic caliper and volumes were calculated (L × W × W/2). Both antibodies and chemotherapeutic agents were administered by intraperitoneal injection. Chemotherapies used in this study were nab-paclitaxel (Abraxane®, NDC 68817-134), irinotecan (Camptosar, 0009-7529) and fluorouracil (Adrucil, 0703-3015).

For tumorigenicity studies, single cell suspensions from control and treated tumors were incubated with biotinylated mouse antibodies (α-mouse CD45-biotin 1:100 dilution and rat α-mouse H2Kd-biotin 1:50 dilution, BioLegend, San Diego, CA) on ice for 30 min followed by addition of streptavidin-labeled magnetic beads (Invitrogen, Carlsbad, CA) to remove murine stromal cells. The remaining human cells in the suspension were collected, counted and diluted to appropriate cell doses, then mixed with a mixture of 1:1 (v/v) FACS buffer and Matrigel and injected subcutaneously in NOD/SCID mice. Tumor growth was monitored for up to 3 months. Cancer stem cell frequency was determined using L-Calc Version 1.1 software program (StemCell Technologies, Inc., Vancouver, Canada).

### RNA Preparation and microarray gene expression

Tumor RNAs were isolated using the RNeasy Fibrous Tissue Mini Kit (Qiagen, Valencia, CA) with DNAseI treatment and were amplified using the Ovation RNA Amplification System V2 (NuGEN, San Carlos, CA). Global gene expression profiles were assayed on Affymetrix HG-U133plus2 human chips. Array background adjustment and signal intensity normalization were performed with GCRMA algorithm for the comparison experiments or frozen RMA algorithm for the baseline PDX model and primary tumor samples in the open-source Bioconductor software (www.bioconductor.org). Genes differentially expressed between the control and 131R10 or combination with irinotecan groups were identified with Bayesian t-test (Cyber-T), which combines student’s t-test with a Bayesian estimate of the intra-group variance obtained from the observed variance of probe sets at a similar expression levels. For experiments with more than two groups, Bayesian one-way ANOVA and Tukey’s HSD post hoc test were performed. We set the Sliding Window Size parameter at 101 and the Bayes Confidence Estimate parameter at 10. The significantly regulated genes were chosen based on the p-value < 0.05 and absolute fold change ≥1.5.

### Targeted Gene Expression

Tumor RNAs were isolated using the RNeasy Fibrous Tissue Mini Kit (Qiagen, Valencia, CA) with DNAse1 treatment. Quantitative real-time PCR was performed either in an ABI 7900HT and analyzed using SDSv2.3 (Applied Biosystems, Foster City, CA) using the comparative Ct method or in a Biomark HD analyzer (Fluidigm). Assays were verified to detect their target genes across a linear dynamic range and were evaluated for any cross-species reactions and genomic DNA detection. Assays did not exhibit any non-specific detection. All gene expression assays were obtained from Applied Biosystems: *LGR5* (Hs00173664_m1), *RSPO3* (Hs00262176_m1), *ZNRF3* (Hs00393094_m1), *Rspo3* (Mm01188251_m1), *Wnt2b* (Mm01306382_m1), *Wnt5a* (Mm00437347_m1), *Acta2* (MM00725412_s1) and *S100a4* (Mm00803372_g1). The PCR primers for human and murine RSPO3 were determined to be species specific so that gene expression could be quantified in both tumor (human) and stromal (murine) cellular compartments using RNA prepared from xenograft tumors.

### *In Situ* Hybridization

*In situ* hybridization (ISH) was performed using RNAscope® by Advanced cell Diagnostics (ACD, Newark, CA). The RNAscope assay was performed on 4-micron thick sections of FFPE tissues using the RNAscope® 2.5 VS Reagent kit. Samples were stained for ISH on Ventana Discovery Ultra instrument using Ventana mRNA Amplification, Pretreatment & DAB Kit (Ventana Medical Systems, Tucson, AZ). RNAscope probes targeting RSPO3 were developed by ACD. The UBC and DAPB probes were developed by ACD and used as a positive and negative control, respectively.

### Molecular Pathology

Immunohistochemistry (IHC) assays were developed and optimized for Phospho-HISTONE H3 (Ser10, pHH3) (9701, Cell Signaling, Danvers, MA) and β-CATENIN (610154, BD Biosciences). FFPE tissue sections were subjected to antigen retrieval and then stained for single or dual IHC on a Ventana Benchmark Ultra instrument using Ventana UltraMap reagents. For dual IHC of pHH3 and β-CATENIN, we visualized pHH3 with Warp Red chromogen following MACH 2 AP-Polymer (Biocare Medical, Pacheco, CA). Sections were then counterstained with hematoxylin and coverslipped. Slides were scanned using an Aperio AT scanner (Leica Biosystems, Buffalo Grove IL).

### Statistical Analysis

Data for PDX tumor measurements are expressed as mean + S.E.M. and differences between groups were quantified with GraphPad Prism7 using repeated measures two-way ANOVA with multiple planned comparisons.

## Electronic supplementary material

Supplementary Information
