# Supplementary material for: RSPO3 antagonism inhibits growth and tumorigenicity in colorectal tumors harboring common Wnt pathway mutations
Source: Sci Rep. 2017 Nov 10;7:15270. doi: 10.1038/s41598-017-15704-y (PMC5681666; doi:10.1038/s41598-017-15704-y)
Supplement: Supplementary file 1 — Supplementary Information [file 41598_2017_15704_MOESM1_ESM.pdf]

Supplementary Information

RSPO3 antagonism inhibits growth and tumorigenicity in colorectal tumors harboring common Wnt pathway mutations

Marcus M. Fischer[1]*, V. Pete Yeung[1]*, Fiore Cattaruzza[1], Rajaa Hussein[1], Wan-Ching Yen[1], Christopher Murriel[1], James W. Evans[1], Gilbert O'Young[1], Alayne L. Brunner[1], Min Wang[1], Jennifer Cain[1], Belinda Cancilla[1], Ann Kapoun[1] and Timothy Hoey[1]

*co-first authors

[1]OncoMed Pharmaceuticals, Inc.
800 Chesapeake Drive
Redwood City, CA 94063 USA

Extended Fig. 1

	VACO6		SNU1411	
	**anti-RSPO3**	**anti-RSPO3 + irinotecan**	**anti-RSPO3**	**anti-RSPO3+ irinotecan**
**RSPO3**	1.26	1.11	1.15	1.05
**AXIN2**	**-4.64**	**-4.35**	**-11.76**	**-2.94**
**ZNRF3**	**-2.57**	**-2.92**	**-4.08**	-1.42
**LGR4**	-1.49	-1.46	1.37	1.03
**LGR5**	**-16.01**	**-12.19**	**-2.8**	**-2.75**
**LGR6**	1	1	1.02	1.45

Bold: Significant probesets with |Fold change| >1.5 and p-value<0.05

Extended Fig. 2

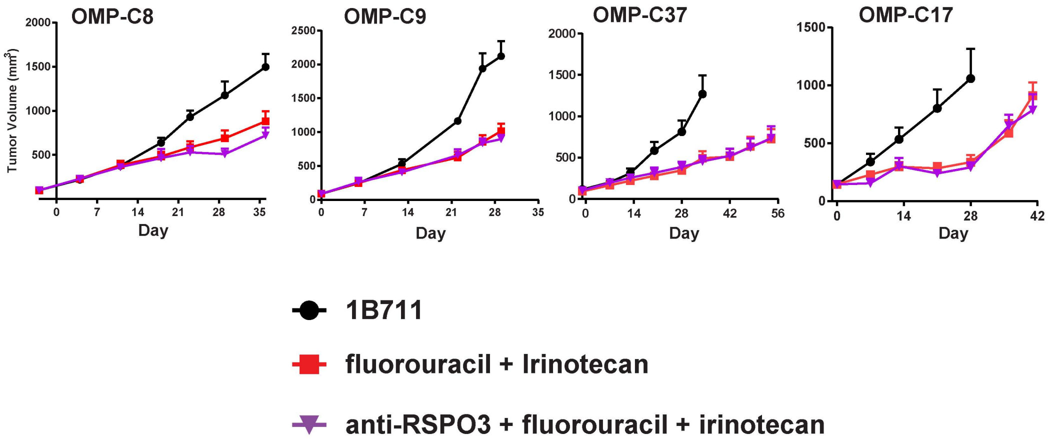

Extended Fig. 3

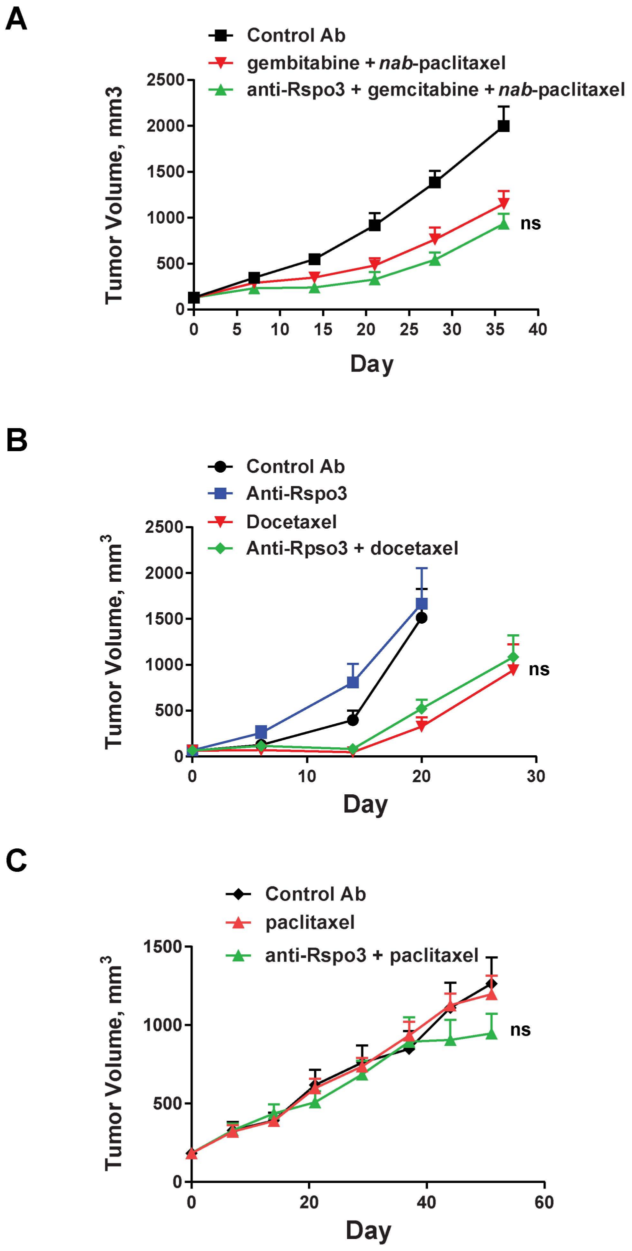

Legends for Extended Figures.

Extended Figure 1. Microarray analysis of tumor RNA from VACO6 and SNU1411 studies. Genes differentially expressed between the control and 131R10 or combination with irinotecan groups were identified with Bayesian t-test, n=3 tumors per group. The significantly regulated genes were chosen based on the p-value <0.05 and absolute fold change ≥1.5.

Extended Figure 2. CRC models treated with anti-Rspo3 and fluorouracil with irinotecan do not respond to therapy. OMP-C8, OMP-C9, OMP-C37, and OMP-C17 were treated with anti-Rspo3 (25 mpk Q2W) dosed on day 1 and fluorouracil (50 mpk Q1W) and irinotecan (5 mpk Q1W) dosed on day 3 and day 10, in two week cycles. n=7-10 per treatment group, expressed as mean + SEM, ns=not significant.

Extended Figure 3. Tumor models without Wnt activating mutations or *RSPO3* expression treated with anti-Rspo3 and chemotherapy do not respond to therapy. A. OMP-PN28 was treated with anti-Rspo3 (25 mpk Q2W) and gemcitabine and nab-paclitaxel. B. OMP-LU33 was treated with anti-Rspo3 (25 mpk Q2W) and docetaxel. C. OMP-OV22 was treated with anti-Rspo3 (25 mpk Q2W) and paclitaxel. ns=not significant.
